# Unraveling the Role of Adiponectin Receptors in Obesity-Related Breast Cancer

**DOI:** 10.3390/ijms24108907

**Published:** 2023-05-17

**Authors:** Giuseppina Daniela Naimo, Alessandro Paolì, Francesca Giordano, Martina Forestiero, Maria Luisa Panno, Sebastiano Andò, Loredana Mauro

**Affiliations:** 1 Department of Pharmacy, Health and Nutritional Sciences, University of Calabria, 87036 Arcavacata di Rende, CS, Italy; giuseppinadaniela.naimo@unical.it (G.D.N.); alessandro.paoli28@gmail.com (A.P.); francesca.giordano@unical.it (F.G.); martina.forestiero@unical.it (M.F.); mluisa.panno@unical.it (M.L.P.); 2 Centro Sanitario, University of Calabria, 87036 Arcavacata di Rende, CS, Italy

**Keywords:** breast cancer, adiponectin receptors, adiponectin, seven-transmembrane receptors, obesity

## Abstract

Obesity has a noteworthy role in breast tumor initiation and progression. Among the mechanisms proposed, the most validated is the development of chronic low-grade inflammation, supported by immune cell infiltration along with dysfunction in adipose tissue biology, characterized by an imbalance in adipocytokines secretion and alteration of their receptors within the tumor microenvironment. Many of these receptors belong to the seven-transmembrane receptor family, which are involved in physiological features, such as immune responses and metabolism, as well as in the development and progression of several malignancies, including breast cancer. These receptors are classified as canonical (G protein-coupled receptors, GPCRs) and atypical receptors, which fail to interact and activate G proteins. Among the atypical receptors, adiponectin receptors (AdipoRs) mediate the effect of adiponectin, the most abundant adipocytes-derived hormone, on breast cancer cell proliferation, whose serum levels are reduced in obesity. The adiponectin/AdipoRs axis is becoming increasingly important regarding its role in breast tumorigenesis and as a therapeutic target for breast cancer treatment. The objectives of this review are as follows: to point out the structural and functional differences between GPCRs and AdipoRs, and to focus on the effect of AdipoRs activation in the development and progression of obesity-dependent breast cancer.

## 1. Introduction

Obesity is a multifactorial metabolic disorder characterized by an abnormal accumulation of adipose tissue and is associated with an increased incidence of several diseases and malignancies [1]. The World Health Organization (WHO) and the National Institute of Health (NIH) have provided accurate guidelines to clinically define an obese subject, according to the body mass index (BMI). Although BMI is the mainstream indicator for diagnosing obesity, it is not sufficient to explain the complexity of obesity status and its related disorders [2]. Particularly, it has been evidenced that body composition and adipose tissue depots distribution also play a relevant role in contributing to metabolic and hormonal abnormalities, which are linked to an increased risk of developing several cancer types, including breast cancer [2]. In this context, it has been observed that the association between breast cancer risk and metabolic dysfunction is independent of BMI. Indeed, individuals with metabolically unhealthy normal weight or overweight/obesity may have an increased risk and incidence of breast cancer compared to metabolically healthy normal-weight individuals [3].

Obesity and breast cancer are both public health issues, and strong evidence correlates obesity with worse disease-free and overall survival [4].

Currently, breast cancer is the most commonly diagnosed cancer and represents the leading cause of cancer-related mortality among women worldwide. According to the latest cancer data accessible from the GLOBOCAN 2020 database, globally breast cancer accounts for 24.5% of all new cancer cases and 15.5% of all cancer-related deaths, with an estimated number of incident cases of 531.086 and 141.765 deaths in Europe [5]. Tumor initiation and development are closely dependent on the cross-talk between breast epithelial cells and the surrounding tumor microenvironment (TME), mainly composed of adipocytes, peripheral immune and inflammatory cells, cancer-associated fibroblasts and microvessels, embedded with various cytokines and chemokines [6].

Among the cellular components of TME, adipocytes are the main actors in regard to the link between obesity and breast cancer risk. Indeed, adipose tissue has been identified as an active organ engaged in the regulation of inflammation, metabolism, and tissue remodeling through the production of many bioactive polypeptides known as adipokines [7].

Obesity is associated with chronic low-grade inflammation, which is related to multiple changes in adipose tissue biology, including dysfunction in adipokines secretion such as adiponectin [1].

A large body of evidence suggests that the infiltration of proinflammatory macrophages into the adipose tissue, resulting in the secretion of inflammatory mediators, contributes to the maintenance of chronic low-grade inflammation, an essential condition to create a suitable TME for breast cancer development, progression, and metastasis [6,8].

These secretory factors act by binding to specific receptors, whose regulation is affected by autocrine, paracrine and endocrine mechanisms. Most of these receptors are cell surface receptors belonging to the seven-transmembrane receptor family. This receptor group consists of two distinct subfamilies, canonical G protein-coupled receptors (GPCRs) and atypical receptors, characterized by a number of peculiar structural and functional properties [9,10]. Although they share a seven-transmembrane architecture, each receptor subfamily presents a unique conformation and the ability to initiate signaling through the engagement of distinct transduction molecules.

There are two main hallmarks for a protein to be classified as a GPCR: the presence of seven α-helices that span the plasma membrane, and the ability of the receptor to interact with a G-protein [9,10].

**Figure 1:**
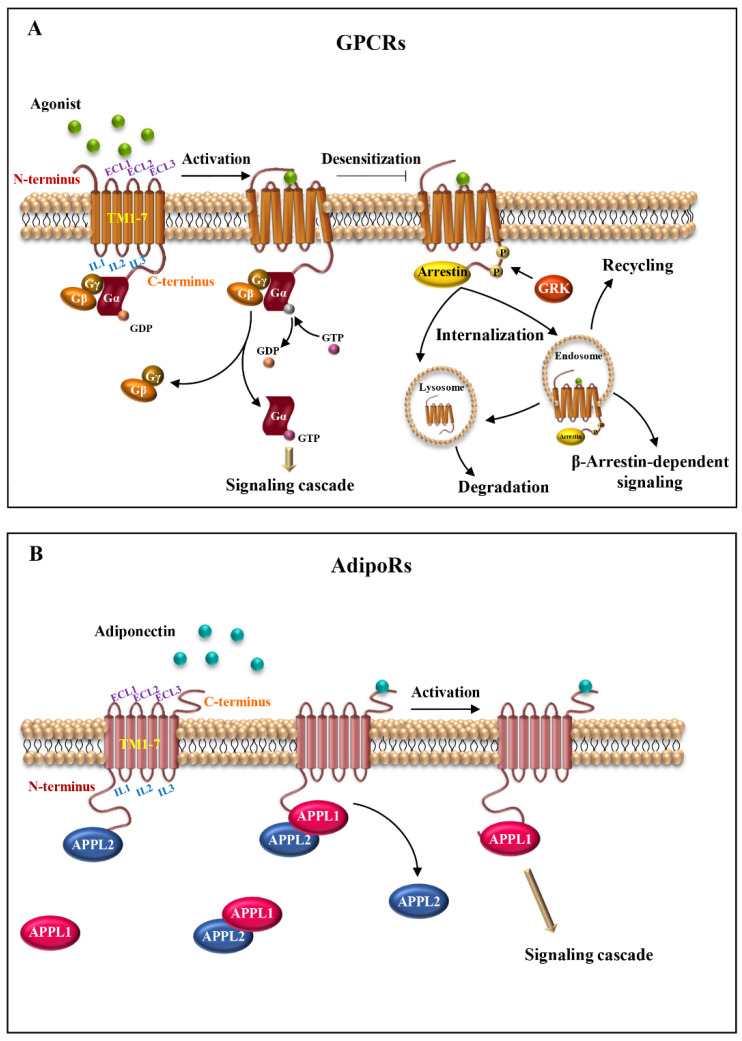
Seven-transmembrane receptor structure and activation. (**A**) Schematic representation of the basic structure of GPCRs. The receptors comprise seven transmembrane helices (TM1-7) connected by intracellular (IL1-3) and extracellular (ECL1-3) loops, with extracellular N-terminus and intracellular C-terminus. The binding of the agonist to the receptor induces conformational changes and activation of heterotrimeric G proteins (αβγ). Next, after the exchange between GDP and GTP, α-subunit dissociates from βγ dimer, initiating signal cascades. (**B**) Illustrative scheme of AdipoRs structure organization. AdipoRs are atypical seven-transmembrane proteins, consisting of seven integral membrane domains (TM1-7) and extracellular and intracellular loops (ECL1-3 and IL1-3, respectively), with an internal N-terminus and an external C-terminus. AdipoRs signaling is mediated through APPL isoforms, APPL1 and APPL2. In the absence of adiponectin, APPL2 binds to the N-terminal domain of AdipoRs or forms an APPL1/APPL2 heterodimer, preventing receptors activation. Conversely, in the presence of adiponectin, APPL2 dissociates from APPL1 and AdipoRs. Adiponectin/AdipoRs interaction promotes APPL1 association and subsequent activation of different signaling pathways.

Structurally, these receptors consist of three parts: the extracellular, the transmembrane (TM) and the intracellular region. The extracellular region contains the N-terminus and three extracellular loops (ECL1-ECL3); the transmembrane region is composed of seven transmembrane domains (alpha-helices, TM1-TM7) that span the plasma membrane in a counter-clockwise manner, forming a recognition and connection unit; the intracellular region presents three intracellular loops (ICL1-ICL3), a short intracellular amphipathic alpha-helix (H8) and the C-terminus [9,10]. The extracellular region regulates the interaction with the ligands; the TM regions bind the ligands and transduce the signal to the intracellular side, causing a conformational change, which allows the interaction with cytosolic signaling proteins (Figure 1A) [11].

These receptors transduce a wide array of signals and regulate many cellular physiological and pathological processes, representing attractive drug targets.

GPCRs detect multiple extracellular signals and activate intracellular responses through the interaction with heterotrimeric G-proteins (α, β and γ subunits), initiating a cascade of G protein-mediated signaling pathways, which are also involved in the desensitization and internalization of the receptor (Figure 1A) [9].

**Figure 2:**
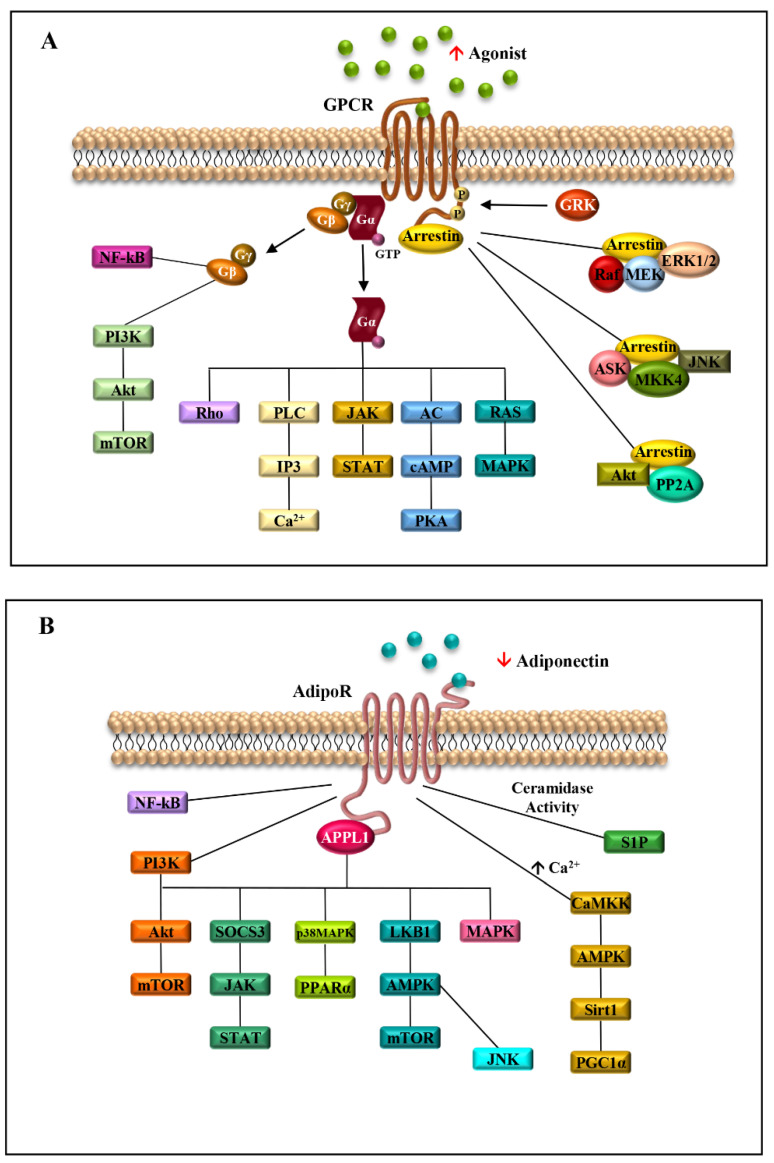
Seven-transmembrane receptors signaling pathways activated in obesity-related breast cancer. Schematic representation of the signaling cascades activated by the binding of agonists to GPCRs (**A**) or adiponectin to AdipoRs (**B**) in obesity conditions. ↑ agonist indicates the high level of GPCR agonists in obesity; ↓ adiponectin shows the reduced level of adiponectin, typical of obese status.

Due to the great heterogeneity and their involvement in a plethora of biological processes, GPCRs take part in the activation of a variety of intracellular signalings, such as ERK1/2, JNK, p38 mitogen-activated protein kinases (MAPKs) and PI3K/Akt (Figure 2) [12].

The seven-transmembrane receptors are key regulator elements in a wide range of physiological processes, including hormonal signal transduction, cell survival, proliferation and differentiation [13].

In the last few years, many studies have demonstrated evidence that different cell types (e.g., immune, normal mesenchymal, stromal and tumor cells) express atypical chemokine receptors (ACKRs) that are seven-transmembrane cell surface proteins. These receptors regulate cell migration in inflammatory, immune and pathological contexts. ACKRs, in contrast to canonical GPCRs, fail to induce the full spectrum of signaling activated by G proteins. This is due to a structural peculiarity in ACKRs represented by the modified DRYLAIV motif within the second intracellular loop. These amino acid substitutions make ACKRs unable to bind to the G protein α subunit and to activate the related downstream signaling and cellular responses [14,15].

A singular case of seven-transmembrane domain receptors, not coupled with G proteins, is represented by the adiponectin receptors, AdipoR1 and AdipoR2 (AdipoRs) [7,16].

Adiponectin is one of the adipokines produced by adipose tissue that has recently attracted more attention for its crucial role in obesity-related disorders [1,17].

In adipocytes, adiponectin is produced as a monomeric protein, which undergoes posttranslational modification into different multimers before secretion into the bloodstream [18]. AdipoRs are ubiquitously expressed with a predominant level of one of the two receptor subtypes depending on the specific tissue. AdipoR1 is highly expressed in adipose tissue, suggesting that adiponectin may exert its biological effects in an autocrine/paracrine manner [7,19]. Paradoxically, in the adipose tissue of obese individuals, AdipoR1 expression is reduced, whereas weight loss elicits its increase [20].

AdipoRs regulate a plethora of biological effects, including insulin-sensitizing, anti-inflammatory, anti-angiogenic and vasodilatory properties [21]. Adiponectin also plays an important role in energy homeostasis by enhancing fatty acid oxidation in skeletal muscle and inhibiting glucose production in the liver [22].

AdipoRs topology is opposite to that of the classical GPCRs, with an internal N-terminus and an external C-terminus, enabling them to interact with G proteins [19]. Indeed, the AdipoRs bind to the adaptor protein containing a pleckstrin homology domain, phosphotyrosine binding domain and leucine zipper motif (APPL). APPL exhibits two isoforms, APPL1 and APPL2, which positively and negatively regulate adiponectin signaling, respectively [23]. In the absence of adiponectin stimulation, APPL2 binds to the N-terminus domain of AdipoRs or undergoes heterodimerization with APPL1, preventing the APPL1/AdipoRs from binding. On the other hand, upon adiponectin binding to AdipoRs, APPL1 dissociates from APPL2, leading to the activation of downstream signal transduction (Figure 1B) [19,24,25,26].

Multiple epidemiological evidence correlates low adiponectin level, characterizing obesity status, with an enhanced risk of cancer development, such as kidney, esophagus, prostate, thyroid, pancreatic, colorectal, breast cancer and many others [27].

In cancer cells, the activation of AdipoRs impacts cell proliferation, survival, apoptosis, migration and invasion, supporting tumor growth and progression [1,27].

Although AdipoRs and GPCRs enroll different intracellular protein effectors, their activation may trigger signaling pathways that mediate similar biological effects in tumor cells.

The objective of this review is to summarize the prevailing knowledge on atypical seven-transmembrane receptors, focusing on the role of AdipoRs in sustaining obesity-related breast cancer growth and progression.

## 2. Adiponectin Receptors and Adiponectin

Many reports validate the involvement of seven-transmembrane receptors in the pathogenesis of several human diseases, such as diabetes, obesity, cardiovascular dysfunctions and malignancies [1,28,29]. Emerging evidence underlines the important role of seven-transmembrane receptors and their signaling in tumor growth and development [2,25,30,31]. In fact, seven-transmembrane receptors control many features of tumor biology, including proliferation, invasion, survival, and metastasis. These receptors are not only expressed by cancer cells but also by different cell types in the tumor microenvironment, such as stromal, vascular, and immune cells [6,32,33].

As seven-transmembrane receptors play an important role in a wide variety of physiological and pathological processes, they are commonly targeted for medicinal therapeutics [25,28,34,35].

Adiponectin receptors, AdipoR1 and AdipoR2, cloned for the first time in 2003, are located at chromosome 1p36.13-q41 and 1 E4, and chromosome 12p13.31 and 6 F1, respectively. The analysis of cDNA encoded in these proteins showed that human and mouse AdipoR1 share 96.8% identity, whereas human and mouse AdipoR2 share 95.2% [36,37].

Similarly to other GPCRs, both AdipoR1 and AdipoR2 are integral membrane proteins, containing seven-transmembrane domains, an N-terminus and a C-terminus. However, their configuration is opposite to GPCRs, with an internal N-terminus and an external C-terminus. This makes AdipoRs unable to interact with G proteins [38,39] (Figure 1B). These peculiarities make adiponectin receptors structurally and functionally distinct from GPCRs [16,39].

Thus, even though AdipoRs and GPCRs families share the same seven-transmembrane domain structure, their sequence homology is weak [38].

In contrast to most of the GPCRs, knowledge of the conformational status of AdipoRs with respect to transmembrane signaling remains to be better defined [9,40].

Thus, structural details about AdipoRs could provide important information on the activation of specific signaling pathways, as well as for the development of specific agonists. For instance, Tanabe and coworkers demonstrated by crystallographic studies that AdipoR1 assumes a dual conformation, consisting of the closed and open forms, related to the signaling mechanisms [40].

AdipoRs mediate the pleiotropic actions of adiponectin, the most abundant fat-derived hormone, whose reduction plays a pivotal role in obesity-linked diseases, including insulin resistance/type 2 diabetes, atherosclerosis and malignancies [21,37].

The gene encoding adiponectin is expressed mainly in adipocytes and is regulated by different transcriptional factors, such as C/EBPs [41], sterol regulatory element binding to protein 1c (SREBP1c) [42] and PPARγ [43]. This adipokine consists of a C-terminal globular domain and an N-terminal collagen-like domain, which makes it structurally similar to complement 1q, belonging to a protein family that forms characteristic multimers [22]. Once synthesized, adiponectin is secreted into the bloodstream in different isoforms: a low-molecular-weight (LMW) trimer, a middle-molecular-weight (MMW) hexamer and a high-molecular-weight (HMW) 12- to 18-mer [37,44,45]. Moreover, a globular form, derived from full-length adiponectin by proteolytic cleavage, has been detected in plasma [37].

The interaction between ligand and receptor takes place through the globular domain of adiponectin and the extracellular portion of the receptors [46]. AdipoRs are unique and ubiquitously expressed, even though they differ from each other, both for the predominance of tissue localization and for their affinity to adiponectin isoforms [7]. In particular, AdipoR1 shows a higher affinity for globular adiponectin and is predominant in the skeletal muscle [7]. In contrast, AdipoR2 exhibits a higher affinity for full-length adiponectin and is most abundant in the liver [19,27]. AdipoRs contain a zinc-binding catalytic site coordinated by three histamine residues located near the inner surface of the plasma membrane [38]. The binding of adiponectin to the zinc-binding motif of AdipoRs triggers the activation of downstream signaling pathways in several target tissues, including skeletal muscle, liver, heart, kidney, and pancreas [47].

The signal transduction is mainly regulated by the adaptor protein APPL1, which interacts with the intracellular region of AdipoRs [48].

The engagement of AdipoRs by APPL1 increases the phosphorylation of adenosine monophosphate protein kinase (AMPK) and p38 mitogen-activated protein kinase (p38 MAPK), and enhances peroxisome proliferator-activated receptor alpha (PPARα) ligand activity, thereby inducing increased fatty acid oxidation and glucose consumption [36]. In particular, AMPK activation also depends on the different mechanisms, such as LKB1 cytosolic localization, driven by APPL1, or phospholipase C/Ca^2+^/Ca^2+^/calmodulin-dependent protein kinase kinase-dependent pathway, triggering an increased release of Ca^2+^ from the intracellular stores [1]. In addition, AdipoRs possess intrinsic ceramidase activity, which leads to a decrease in intracellular ceramide, a sphingolipid involved in insulin resistance, atherosclerosis, inflammation, and cell death (Figure 2B) [47]. The adiponectin/AdipoRs complex also stimulates fatty acid oxidation in skeletal muscle, inhibits glucose production in the liver, prompting an improvement in energy homeostasis, and mediates anti-inflammatory and anti-apoptotic actions in different cell types [46].

Obesity not only leads to a decrease in plasma adiponectin levels but also to an altered AdipoRs expression, modulating adiponectin sensitivity and causing insulin resistance with consequent hyperinsulinemia [49]. Moreover, a decreased expression of AdipoRs in the skeletal muscle of type 2 diabetic patients has also been found [50]. Therefore, further studies are needed for a deeper understanding of the role of the adiponectin/AdipoRs complex in obesity-associated diseases and in the identification of molecular targets to be used for therapeutic purposes.

### Signaling Pathways Activated by Seven-Transmembrane Receptors in Breast Cancer

Despite AdipoRs and GPCRs specifically exploiting different transduction proteins to initiate signaling cascades, they activate downstream signaling involved in different proliferative and pro-inflammatory cytokine actions.

Chronic inflammatory status, supported by cytokines, is an important aspect of the pathogenesis of obesity-related breast cancer [51]. It is largely known that obese individuals are affected by more aggressive breast tumors, with an increased risk of recurrence and higher mortality [52]. Indeed, in an obese adipose tissue, the imbalance of cytokine production creates a chronic inflammatory microenvironment that promotes breast tumor cell motility, invasion, and epithelial-mesenchymal transition, enhancing the tumor cells’ metastatic potential [7,52,53,54]. Moreover, in this scenario, different cytokines may act through autocrine and paracrine mechanisms to modulate the activation of specific receptors, influencing breast tumor behavior.

In recent decades, growing evidence has highlighted the critical role of adiponectin/AdipoRs signaling as one of the main prediction markers of breast tumor clinical outcomes in obese patients. Indeed, in obesity-related breast cancer, the activation of AdipoRs affects cell proliferation, survival, apoptosis, migration and invasion, endosomal trafficking, metabolism and chromatin remodeling [7,48,55,56,57,58].

**Figure 3:**
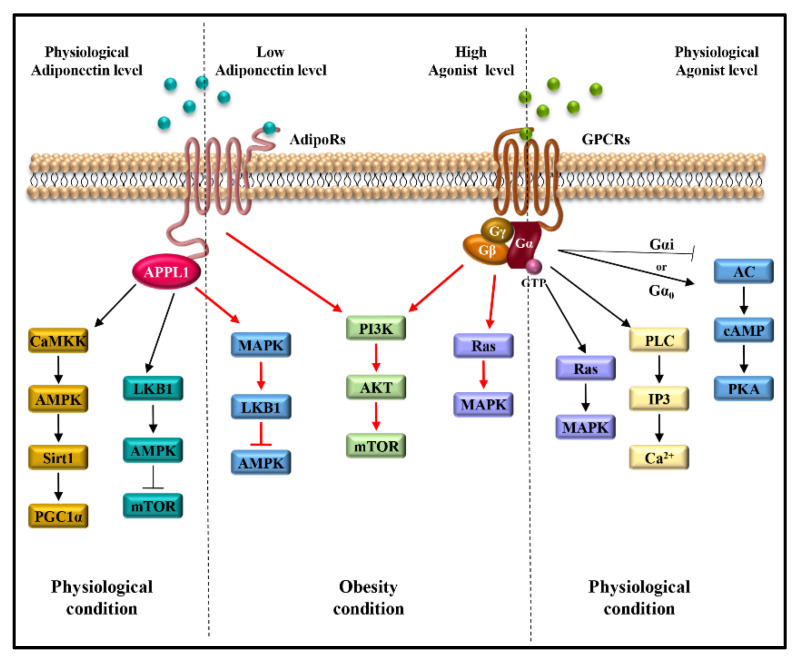
Signaling pathways activated by atypical (AdipoRs) and classical (GPCRs) seven-transmembrane receptors in obesity-related breast cancer. Red lines indicate the shared signaling activated by low concentrations of adiponectin and by high levels of GPCRs agonists, which characterize obesity status, and favor breast cancer growth and progression.

The main transduction pathways activated by adiponectin/AdipoRs/APPL1 axis include JAK/STAT, LKB1/AMPK/mTOR, AMPK/Sirt1/PGC1α, NF-kB, JNK and MAPK, sustaining breast tumor development and progression through the induction of cell proliferation and survival, cell cycle progression and anti-apoptotic response [18,59] (Figure 2B). Moreover, the stimulation of AdipoR1 and AdipoR2 induced by adiponectin leads to the activation of AMPK and PPARs, respectively, involved in the regulation of inflammatory responses, and lipid and glucose metabolism [60]. In the context of TME, the stimulation of AdipoR1 may potentiate the effect of pro-inflammatory cytokines. For example, in the TME, the high levels of CXCL12, which binds its seven-transmembrane receptor, CXCR4, and the low levels of adiponectin may concomitantly contribute to mediating cancer cell growth and progression [61,62,63,64]. Indeed, the adiponectin/AdipoR1 complex and the CXCL12/CXCR4 axis trigger the activation of several transduction signalings—Ras/MAPK, PI3K/AKT/mTOR, JNK/p38MAPK, Jak/STAT, NF-kB, PLC and MAPK—involved in cell survival, cell proliferation and migration [65,66,67,68,69]. Moreover, the inhibition of adenylate cyclase and consequent cAMP formation, induced by the CXCL12/CXCR4 complex, favors cancer cell proliferation [70,71]. Thus, the activation of all these mentioned pathways may create a suitable TME more prone to sustain breast cancer growth and progression (Figure 3).

## 3. Breast Cancer and Obesity

Breast cancer consists of a heterogeneous group of malignant tumors that are different in morphology, gene expression patterns, tumor behavior, clinical management, and prognosis [72].

The traditional, histological, pathological staging and tumor differentiation classifications provide diagnostic and prognostic information that is still important but not sufficient to satisfy the need for personalized therapies [72].

Indeed, tumors that at first glance are similar in morphology and stage actually may have a variable prognosis and therapeutic responses.

Thus, due to breast cancer’s biological and clinical complexity, a more accurate tumor classification is critical for individualized patient care [72].

According to the current molecular classification, breast cancers are grouped as follows: luminal A: ER+ and/or PR+, HER2- and low Ki-67 (<14%); luminal B: ER+ and/or PR+, HER2+ or HER2- and high Ki-67 (>14%); HER2+: ER-, PR- and HER2+; and basal-like (BLBC): ER-, PR-, HER2- (triple negative), plus CK 5/6+ and/or EGFR+ [72]. More recently, PD-L1 has been recognized as a predictive marker for the potential response to immunotherapy in the treatment of positive advanced/metastatic triple-negative breast cancer [73].

In addition, to better understand the heterogeneous nature of breast cancer, close attention is being paid to protective and negative factors, which may affect the development and progression of the disease.

Obesity/overweight is the main lifestyle-related risk factor associated with increased comorbidities and premature deaths [2,74].

A meta-analysis of 82 follow-up studies showed a 35% higher risk of breast cancer mortality in obese women than in their normal-weight counterparts, ascribing obesity to a relevant role as a risk factor for breast tumorigenesis [75].

The mechanisms connecting obesity with cellular pathways contributing to breast tumorigenesis include the hypertrophic and hyperplastic status of adipocytes, which entail an altered adipokine, cytokine and hormone production and secretion, as well as a modulation of their own receptor expression [4]. Moreover, the dysfunctional adipocytes engulf vascular stroma, sustaining local hypoxia that leads to the recruitment of inflammatory cells.

Many reports have addressed the critical role of these factors in sustaining low-grade chronic inflammation and a favorable tumor microenvironment in the development and progression of several obesity-related neoplasias [2,24,70]. In addition, adipose tissue, which acts as a source of estrogens, may contribute to obesity-associated hormone-responsive cancer growth. Moreover, the reduction in adiponectin levels in obese subjects has drawn particular attention for its paracrine and autocrine influence on the mammary epithelium.

Growing evidence correlates low adiponectin levels with a more aggressive breast cancer phenotype, characterized by a higher histological grade, large tumor size, lymph node metastasis and increased mortality [4,22,76]. In this scenario, the action of adiponectin on the tumor microenvironment occurs in the context of altered metabolic homeostasis due to adipocytes dysfunction and the activation of epigenetic pathways [70].

The correlation between tumor growth/progression and low adiponectin levels mainly depends on the disruption of adiponectin signaling mediated through AMPK, which normally inhibits proliferative pathways in non-tumoral cells [11].

Interestingly, in obesity, low levels of adiponectin are associated with altered levels of AdipoRs both in breast cancer cells and tumor microenvironment cell components.

Thus, these findings, due to the close interaction between mammary epithelial cells and adipose tissue, address how any imbalance in the hormonal milieu affects not only the initiation and progression of breast cancer but could also affect its response to therapies [76].

### 3.1. Adiponectin Receptors in Obesity-Related Breast Cancer

Many studies demonstrated that adiponectin and its receptors are expressed both in normal and neoplastic breast epithelial cells. However, conflicting data showed no differences between AdipoR1 and AdipoR2 expression between normal breast epithelial cells and breast cancer cells [77]. Thus, further studies are needed to clarify this issue.

Interestingly, adiponectin receptors are known to be differently expressed in breast cancer cell lines independent of ERα status, with a higher expression of AdipoR1 in MCF-7, T47D and MDA-MB-231 cells, and a higher level of AdipoR2 in MDA-MB-361 cells. Although AdipoRs expression was markedly increased in breast tumor tissues compared with control tissues [78], it has been shown that the high expression of AdipoR2 in breast cancer tissue is significantly and positively associated with vascular and lymph vascular invasion [27].

Reports have revealed that AdipoRs in breast cancer cell lines are also modulated by adiponectin [19,24,27], the levels of which were significantly downregulated in cancer patients compared to control subjects. Particularly, the low adiponectin serum levels upregulate AdipoR1 expression in breast cancer tissue as a kind of feedback loop [79].

Interestingly, the downregulation of AdipoR1 expression was observed in the epithelial cells of pre-invasive ductal carcinoma in situ (DCIS) compared to cells of adjacent invasive breast cancer [79,80]. These findings suggested that the loss of AdipoR1 favors the progression of the pre-invasive lesion [79]. On the other hand, it has been shown that decreased adiponectin serum levels and increased AdipoR1 and AdipoR2 expression occur in response to the development of diet-induced obesity in mice [81]. However, it is worth mentioning that another event increasing the incidence of breast cancer may come from ADIPOQ and/or ADIPOR1 single nucleotide polymorphisms [82].

Convincing evidence has demonstrated that low adiponectin concentrations affect breast cancer cell growth both in vitro and in vivo. Specifically, low adiponectin levels are induced by the formation of a multiprotein complex, where AdipoR1 cross-talks with ERα, c-Src and IGF-1R, converging with MAPK activation and increasing cell proliferation [58]. These results suggest a pivotal role of ERα in the adiponectin response. Indeed, in ERα-positive breast cancer cells, it has been demonstrated that among the mechanisms through which low adiponectin levels stimulate cell proliferation, there is the upregulation of Cyclin D1 and the inhibition of LKB1/AMPK/mTOR signaling [57,83]. On the contrary, in ERα-negative breast cancer cells, the activation of anti-proliferative and pro-apoptotic signaling pathways has been observed [57,83,84,85,86,87]. Although adiponectin has been conventionally considered an anticancer agent, all these data ascribe an ERα signaling amplifier role to this adipokine, favoring breast tumor growth and progression.

### 3.2. Adiponectin Receptors in Breast Tumor Microenvironment

**Figure 4:**
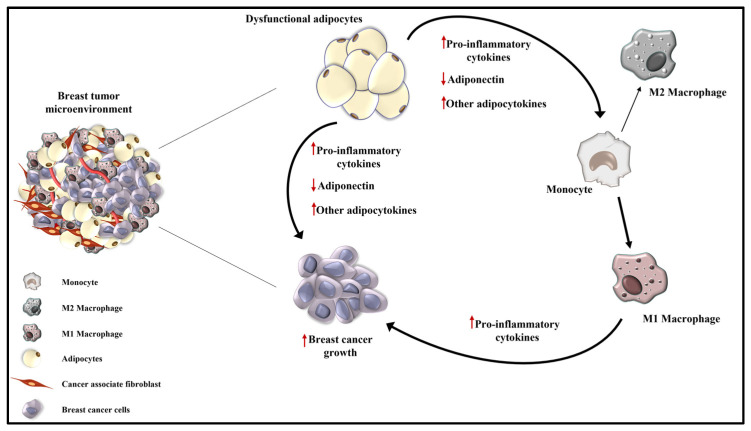
Schematic representation of tumor microenvironment in obesity-related breast cancer. Obesity is characterized by a low-grade inflammatory status sustained by an altered adipocytokines secretion by dysfunctional adipocytes and by an increased infiltration of M1 polarized macrophages that release pro-inflammatory cytokines. All this creates a suitable TME more prone to sustain breast cancer growth and progression. ↑ indicates the increased production of pro-inflammatory cytokines and adipocytokines; ↓ represents the reduced level of adiponectin.

It is widely recognized that tumor development and progression depend on epithelial tissue and the surrounding microenvironment. TME is a highly complex and heterogeneous intricate network, mainly composed of tumor cells, immune and inflammatory cells, cancer-associated fibroblasts and adipocytes, embedded with different factors, such as cytokines, chemokines, growth factors, soluble receptors and exosomes (Figure 4) [6].

Recent evidence has highlighted the existence of a dynamic interaction between breast cancer and the cellular components of TME that drives remodeling of extracellular matrix, neovascularization and epithelial cell transition to stem cells [88]. Thus, by reshaping the TME through the identification of specific molecular targets may represent a cancer treatment strategy.

Adipocytes are considered to be essential components of breast cancer TME [6,89]. In vitro, in vivo and clinical data demonstrate that breast tumor cells significantly affect the surrounding adipocytes, whose derived factors change during tumor progression [6].

Growing evidence has demonstrated that breast cancer progression may be promoted by the interplay of invasive cancer cells with peritumoral adipocytes, referred to as cancer-associated adipocytes (CAAs) [90]. Indeed, breast cancer cells induce the differentiation of normal adipocytes into CAAs, which, compared to mature adipocytes, have smaller cell sizes, irregular shapes, and smaller lipid droplets [91,92]. Furthermore, CAAs are characterized by decreased differentiation markers of mature adipocytes and a more aggressive secretome depending on the overexpression of inflammatory cytokines and proteases, aberrant secretion of adipokines (such as leptin and adiponectin), and promotion of metabolic reprogramming of breast cancer cells [6,90,93].

The bidirectional interaction between breast cancer cells and CAAs triggers the shaping of the TME, leading to the acquisition of an invasive phenotype characterized by proliferation, angiogenesis, tumor dissemination, invasion and metastasis by a less physical barrier.

Notably, it has been demonstrated that there is a significant reduction in adiponectin levels in adipose tissue of breast cancer patients compared to normal breast adipose tissue. On the contrary, the mRNA expression levels of AdipoRs are significantly increased in CAAs [6].

In obesity, a reduction in the adiponectin level is related to DNA methylation of its promoter region in adipocytes. Moreover, low adiponectin expression, in turn, induces an increase in AdipoR2 gene methylation, as reported in humans and obese mice models, suppressing adiponectin binding and action. On the other hand, it has been reported that obesity does not affect AdipoR1 methylation [6,94].

Cicekdal MB and coworkers demonstrated a high methylation level of AdipoR1 in mammary tumor virus-transforming growth factor-α female mice that underwent a chronic energy restriction compared to ad libitum mice [95].

Overall, similarly to mammary adipocytes in obese patients, the reduction in adiponectin secretion by CAAs has been recognized as a crucial event in stimulating breast cancer progression [96].

Moreover, CAAs, once involved in a complex inflammatory process, contribute to the development of drug resistance, which is a crucial driver of tumor progression.

The establishment of chronic low-grade inflammation depends on the increased recruitment of macrophages, distinct in two subpopulations: the pro-inflammatory M1-polarized macrophages and the anti-inflammatory M2 type [97].

More evidence suggests that in lean subjects, the M2 macrophage type is prevalent, while in obese individuals, the M1 type predominates, addressing an M2 to M1 phenotypic switch in adipose tissue in obesity [98,99]. The prevalence of M1 macrophages in the breast cancer microenvironment is also sustained by the recruitment of additional circulating macrophages characterized by a high M1 gene expression that forms inflammatory loci called crown-like structures [1].

Recent data indicate that, through its receptors, different adiponectin isoforms induce isoform-specific responses in human monocytes. Particularly, LMW adiponectin reduces IL-6 release and stimulates IL-10 secretion in differentiated macrophages, favoring anti-inflammatory effects, while HMW increases the secretion of IL-6 in human monocytes, mediating pro-inflammatory responses [100].

Nevertheless, the regulation of AdipoRs expression in monocytes and macrophages deserves to be further explored.

Many studies have suggested the involvement of different hormones and macrophage polarization processes in the modulation of AdipoRs levels in monocytes and macrophages.

Particularly, reports reveal that AdipoRs expression is increased by nuclear hormone receptor ligands, such as LXR and PPAR [101].

Moreover, it has been observed that AdipoR1 is more expressed than AdipoR2 in monocytes, while its expression decreases in M1 macrophages, indicating an important role of macrophage differentiation and polarization in the regulation of the AdipoRs expression.

In M1 polarized macrophages, adiponectin restores AdipoR1 and AdipoR2 levels compared to those in nonpolarized macrophages, thus mediating an inflammatory response. On the contrary, M2 polarized macrophages exhibited higher AdipoRs expression than non-polarized macrophages, regardless of adiponectin stimulation [102].

These data provide important evidence of macrophage polarization in AdipoRs expression and adiponectin-mediated inflammatory response in the breast TME, which profoundly affects cancer progression.

## 4. Conclusions and Future Directions

As breast cancer is the most common cancer and the leading cause of mortality in women worldwide, there is a compelling need to design new therapeutic strategies for the personalized treatment of this malignancy. Tumor initiation and development are affected by the cross-talk between epithelial cells and the surrounding TME components [6]. The chronic low-grade inflammation that characterizes TME in obesity is sustained by immune cell infiltration and altered expression and secretion profiles of inflammatory mediators and their receptors in dysfunctional adipocytes [33]. In this scenario, the decreased adiponectin secreted levels and the dysregulated expression of AdipoRs in breast tumor cells and TME cell components are gaining increasing attention to understand the molecular mechanisms that drive the growth and progression of breast cancer, as well as to identify new therapeutic targets [79,81,101,102]. Particularly, the remodeling of TME is becoming a promising candidate for the development of innovative breast cancer treatment strategies. Indeed, several therapeutic approaches targeted to increase the assembly and secretion of adiponectin have been investigated [1,25]. However, due to adiponectin’s multimeric structure, short half-life and high serum concentrations, it is difficult to obtain benefits from adiponectin replacement therapy.

Thus, the design of AdipoRs agonists aimed at potentiating adiponectin action, making it able to antagonize pro-inflammatory signaling in the TME, represents an attractive therapeutic strategy.

Therefore, the current druggable options focus on the development of orally active small-molecule agonists for AdipoRs. Among these, the ADP355 molecule is the pioneer of the first class of AdipoR1 agonists that reproduces globular adiponectin anti-proliferative activity through the regulation of different signals, such as AMPK, Akt, STAT3 and ERK1/2 [103,104]. Three other peptides designed to mimic adiponectin actions are BHD1028, BHD43 and BHD44, which show a higher stability, solubility and affinity for AdipoR1 compared to ADP355 [105]. Currently, AdipoRon, an oral non-peptidic AdipoRs agonist, is extensively studied due to its adiponectin replacement therapy potentiality. AdipoRon’s main functions are through the activation of AMPK and PPARγ pathways in obesity-related disorders [106]. Despite these molecules being promising candidates in preclinical studies, the AdipoRs agonists listed in this study are currently unavailable for human clinical trials.

In conclusion, the design of innovative AdipoRs agonists and/or the refinement of those that are already available could extend the therapeutic strategies for the treatment of obesity-related breast cancer.
